# After 2015: infectious diseases in a new era of health and development

**DOI:** 10.1098/rstb.2013.0426

**Published:** 2014-06-19

**Authors:** Christopher Dye

**Affiliations:** Office of the Director General, World Health Organization, Avenue Appia, 1211 Geneva 27, Switzerland

**Keywords:** Millennium Development Goals, Universal Health Coverage

## Abstract

Running over timescales that span decades or centuries, the epidemiological transition provides the central narrative of global health. In this transition, a reduction in mortality is followed by a reduction in fertility, creating larger, older populations in which the main causes of illness and death are no longer acute infections of children but chronic diseases of adults. Since the year 2000, the Millennium Development Goals (MDGs) have provided a framework for accelerating the decline of infectious diseases, backed by a massive injection of foreign investment to low-income countries. Despite the successes of the MDGs era, the inhabitants of low-income countries still suffer an enormous burden of disease owing to diarrhoea, pneumonia, HIV/AIDS, tuberculosis, malaria and other pathogens. Adding to the predictable burden of endemic disease, the threat of pandemics is ever-present and global. With a view to the future, this review spotlights five aspects of the fight against infection beyond 2015, when the MDGs will be replaced by a new set of goals for poverty reduction and sustainable development. These aspects are: exploiting the biological links between infectious and non-infectious diseases; controlling infections among the new urban majority; enhancing the response to international health threats; expanding childhood immunization programmes to prevent acute and chronic diseases in adults; and working towards universal health coverage. By scanning the wider horizon now, infectious disease specialists have the chance to shape the post-2015 era of health and development.

## 1. The epidemiological transition

**Figure 1.**
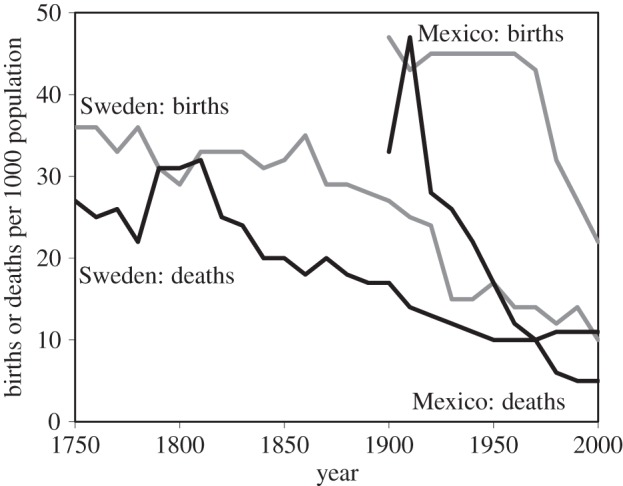
The demographic transitions in Sweden (1750–2000) and Mexico (1900–2000), which are interlinked with the epidemiological transitions in these two countries. The fall in mortality, at first mainly from childhood infectious diseases, is followed by a decline in fertility, producing growing, ageing populations afflicted mainly by chronic, non-infectious diseases. Adapted from reference [2].

The epidemiological transition, interlinked with the demographic transition, provides the central narrative of global health [1]. The transition begins with a fall in the death rate, mainly from acute infectious diseases of childhood. As a higher proportion of children survive to adulthood, parents choose to have smaller families, but the decline in fertility lags behind the decline in mortality. Through time, infectious diseases of childhood are replaced by the chronic, non-infectious diseases typical of adulthood in larger, older populations. In Western Europe, North America and other parts of the industrialized world, this transition has taken place over centuries. In some low- and middle-income countries, such as Mexico, there have been substantial reductions in mortality and fertility in just a few decades (figure 1) [2].

**Figure 2.**
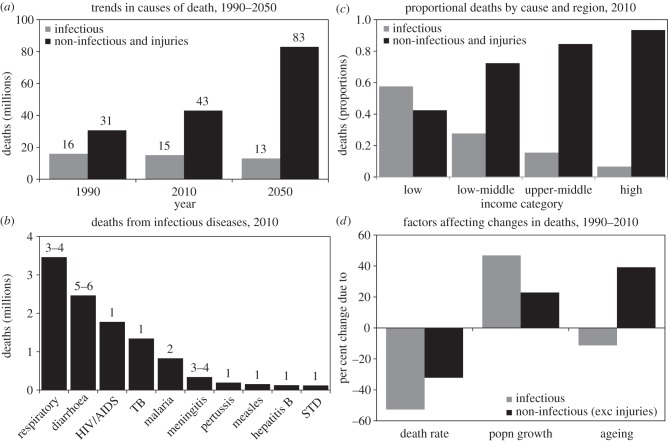
Deaths from infectious diseases (plus maternal and nutritional disorders) and non-infectious diseases (including injuries) worldwide, 1990–2050. (*a*) Estimated deaths in 1990, 2010 and 2050. (*b*) Top 10 causes of death from infectious diseases in 2010. Figures above the bars are the numbers of pathogens causing the majority of deaths from each disease. (*c*) Proportion of deaths due to infectious and non-infectious diseases in low, low-middle, upper-middle and high-income countries in 2010 (World Bank classification). (*d*) Factors affecting percentage changes in the numbers of deaths worldwide, 1990–2010. The fall in death rates *per capita* (left) is offset by population growth (especially deaths from infectious diseases, centre) and ageing (especially deaths from non-infectious diseases, right). Data from references [3,4].

However, nowhere in the world have infectious diseases yet become a negligible cause of illness and death. Worldwide, the number of deaths caused by pathogens and parasites is falling slowly (figure 2*a*). In 1990, an estimated 16 million people died from infections (plus maternal and nutritional disorders). In 2010, the number of deaths had fallen only to 15 million (a decline of only 1% per year). And the World Health Organization (WHO) forecasts 13 million deaths attributed to these causes in 2050 [3]. The fall in the number of people who died between 1990 and 2010 was small, because the drop in mortality *per capita* (−53%) was offset by population growth (47%) [4]. The majority of these deaths have been and will be caused by just a few pathogens: among the 1400 or so recognized human pathogens and parasites [5], two-thirds of deaths from infections in 2010 were caused by around 20 species, mainly bacteria and viruses (figure 2*b*). Set against the slow rate of decline in deaths from infections, the number due to non-infectious diseases has been steadily rising: there were 31 million deaths from non-infectious causes (including injuries) in 1990 and 43 million in 2010 (an increase of 1.6% per year). That number is expected to rise to 83 million in 2050 [3].

Although low-income countries face a rising burden of cardiovascular diseases, cancers, injuries and other non-infectious conditions, infections still caused the majority of deaths in 2010 (figure 2*c*). In addition, these statistics on infectious diseases, which count deaths mostly from endemic infections, exclude the threat of epidemics and pandemics. In short, the epidemiological transition is, so far, only a partial transition. And on present trends, the ‘double burden’ of infectious and non-infectious diseases in low- and middle-income countries is set to remain a double burden for decades to come [6–9].

Since year 2000, attempts to accelerate the decline of major infections have been guided by the Millennium Development Goals (MDGs; especially MDG 6), spearheaded by the assault on HIV/AIDS, tuberculosis (TB) and malaria. As described in §2, the drive to achieve the MDGs has itself been a transition in global health: the marked growth in financial support and the proliferation of health agencies have changed the business of international health beyond recognition.

## 2. The global health transition

**Figure 3.**
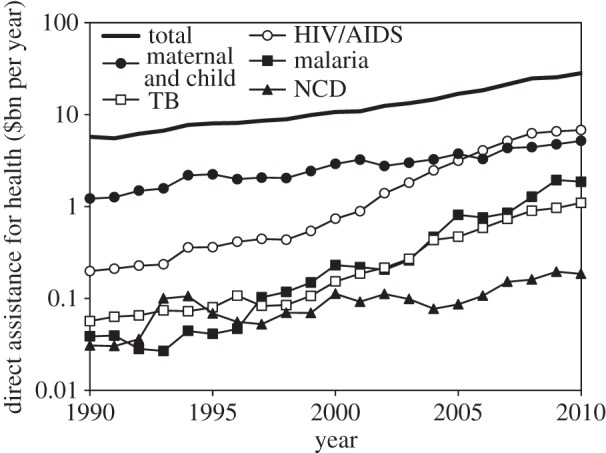
Trends in direct financial assistance for health, 1990–2010, measured in US$ billions per year (log scale), with five of the principal areas of investment. NCD, non-communicable (non-infectious) diseases. Data from reference [10].

While much of the current debate about funding for health is focused on the consequences of an economic recession that began in 2007, the longer term retrospective is far more positive. Over the 20 year period 1990–2010, but especially since 2000, development assistance for health (DAH) has increased nearly fivefold, reaching at least US$27 billion in 2010 [10] (figure 3). This large injection of cash from wealthy countries has supported all areas of health, but particularly the control of HIV/AIDS, malaria and TB. It has led to a proliferation of donors, global funds and partnerships, non-governmental and civil society organizations, philanthropists and commercial investors, and heightened interest from the pharmaceutical industry [11]. Within the past decade, domestic expenditure on health *per capita* has also increased in virtually all countries [12]. This period of rapid expansion has now ended, but a return to the funding patterns of the 1990s seems unlikely.

The huge increase in investment has underpinned some notable achievements. Regarding drug treatment, the development and deployment of antiretroviral drugs for HIV/AIDS is one of the public health successes of the past two decades. By 2012, there were more than 10 million HIV-positive people on antiretroviral treatment (ART), and the cost of drugs had fallen to as little as US$100 per year in sub-Saharan Africa [13]. ART has dramatically restored life expectancy, and prevents, with high-efficacy, viral transmission from mothers to children and between sexual partners [14–16].

On vaccination, the Global Polio Eradication Initiative cut the number of infections by 99% between 1988 and 2013, preventing paralysis in five million people and, remarkably, eliminating the disease from India in 2011. Between 2000 and 2008, measles vaccination reduced deaths by more than 70%. And immunization against maternal and neonatal tetanus has eliminated infection in 20 of 58 high-risk countries [17]. More recently, following WHO pre-qualification of a high-efficacy meningococcal A conjugate vaccine (PsA-TT, commercially known as MenAfriVac), 100 million people were vaccinated across the African meningitis belt between 2010 and 2012 [18–20].

Why, then, has the control of infectious diseases not been more successful? The explanation is a mix of failures to develop and deliver effective technology, both new tools and old. On drug treatment, resistance to antibiotics is becoming more frequent, and yet there is a dearth of new antibiotics. Only two new classes of anti-bacterial drugs (cf. antivirals, such as ART) have been developed and introduced into clinical practice in the past 50 years—the oxazolidinones represented by linezolid, and the lipopeptide antibiotic daptomycin to treat *Staphylococcus* and other Gram-positive bacterial infections [21,22]. On immunization, although the potential impact of a new TB vaccine is enormous [23,24], and even though there are now more candidate TB vaccines than ever before [25], no vaccine with greater efficacy than Bacille Calmette Guérin (BCG) has yet emerged [26]. Likewise, no candidate vaccine for HIV/AIDS or malaria has proved efficacious enough to reach public health practice (although the RTS,S malaria vaccine could be licensed soon [27]).

Even where effective tools already exist, the time taken to deploy them can be years or decades from the point of regulatory approval [28]. Although the WHO strategy for TB treatment has been adopted by every country, implementation has been compromised by the reach of public health systems and by the poor quality of care in private practice [29]. The 10 million HIV-positive people receiving ART in 2012 represented only two-thirds of the 15 million targeted [13]. Drugs to treat helminth infections have been donated in large quantities by pharmaceutical companies, and yet the proportion of eligible children receiving treatment is still far below target. For instance, praziquantel, a one-dose, high-efficacy, preventive or curative treatment for schistosomiasis, was provided to 28 million people in 2011, only 12% of the 240 million people eligible [30]. Furthermore, the treatment of helminth infections is still not closely tied to improved hygiene and sanitation, which would contribute greatly to interrupting transmission [31].

As stated in §1, the limiting factors in infectious disease control are exacerbated by population growth. As long as the burden of infectious disease remains high in low- and middle-income countries, fertility is also likely to remain high. By comparing age-standardized numbers of deaths in 1990 and 2010, figure 2*d* shows how the benefits of falling mortality rates *per capita* (left), enhanced by ageing (right), are counterbalanced by population growth (centre). Ageing, owing to falling fertility, helps to reduce deaths from infections, because most victims are young children.

By contrast, for non-infectious diseases, falling death rates *per capita* are offset mainly by ageing (figure 2*d*, right). Indeed, the global shift towards non-infectious diseases and injuries as leading causes of death is being driven by population growth and ageing, and not by increases in age- and cause-specific death rates [4].

As the 2015 deadline for achieving the MDGs draws closer, vigorous debate is underway about the next set of goals for health and development. Beyond 2015, targeted disease control programmes will continue to be essential. However, a new set of goals will put health in general, and infectious diseases in particular, in the wider context of poverty reduction and sustainable development. Targets will be set for agricultural and urban development, gender equality, education, health, food, water and sanitation, climate change, energy, employment and the management of natural resources [32–34]. The transition through 2015 will bring new challenges, but also new opportunities, for infectious disease control. The following sections highlight five aspects of the fight against infection in the post-MDGs era.

## 3. Linking infectious and non-infectious diseases

A strength of the Global Burden of Disease (GBD) project has been to insist that the number of deaths classified by primary (‘underlying’) cause adds up to the total number of deaths in the world each year [35]. Coherent accounting has helped to eliminate duplications where, for example, deaths among TB cases that are also HIV-positive might have been counted as deaths from both TB and HIV/AIDS. The disadvantage, however, of assigning one death to one cause is that contributing causes of death may be overlooked. The interactions among contributing causes of death are potentially important in explaining epidemiological patterns—the distribution of cases and deaths by person, place and time—and therefore in devising methods for control.

For infectious diseases, whether exposure to a pathogen leads to the establishment of infection and to illness, and the outcome of a course of illness depend on the nature of the pathogen and associated risk factors. Some of these risk factors are chronic, non-infectious diseases. As attention in public health turns to the mounting burden of chronic diseases [36], greater visibility is being given to the biological interactions between the two groups, and to the opportunities to coordinate control efforts.

**Figure 4.**
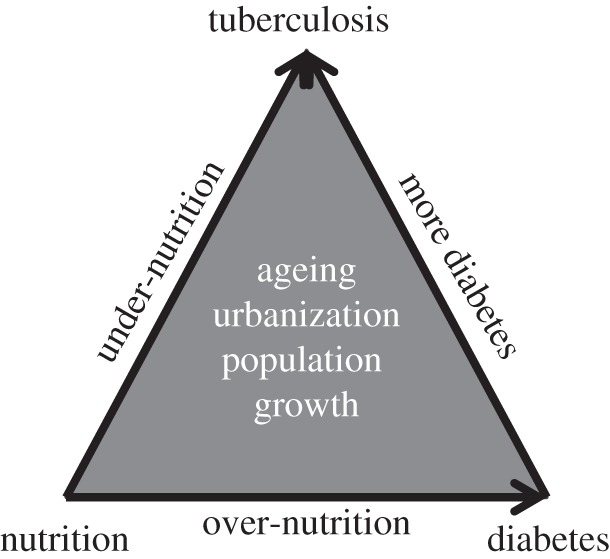
The triangular relationship between nutrition, diabetes and tuberculosis, as investigated in reference [37].

One example arises from the triangular relationship between nutrition, diabetes and TB [37]. Under-nutrition increases the risk of TB by approximately 14% for every unit reduction in body mass index (BMI) [38] (left side of the triangle in figure 4). Individuals with greater BMI are less likely to develop active TB, even to the point of obesity (base of triangle). But overweight is a risk factor for diabetes, which is also associated with a higher risk of TB [39,40] (right side of triangle). The biological basis of these interactions is not fully understood. The implication, however, is that better nutrition and the management of diabetes could contribute to the prevention of TB. Reciprocally, the clinical management of TB presents an opportunity to identify people who are undernourished or who have diabetes. To illustrate, if the prevalence of diabetes in a population is 10% and the relative risk for TB among diabetics is 3, then the prevalence of diabetes among TB cases will be (0.1 × 3)/[1 + 0.1 × (3–1)] = 0.25. So, 25% of TB cases come from 10% of the population. The interaction is a chance to improve the efficiency of both infectious and non-infectious disease control programmes [41–43].

Diabetes and under-nutrition are just two among numerous risk factors for TB. In considering the best approach to TB control, their importance must be placed alongside other kinds of risk, for example over-crowded housing, the poor awareness of symptoms among patients, the lack of access to diagnostic and treatment facilities, the prohibitive cost of drugs to treat multidrug-resistant strains, medical malpractice, broken drug supply chains and patients not completing their treatment. Although these shortcomings are not usually thought to be risk factors [44], they account for a large proportion of the avertible burden of disease. On a post-2015 agenda for poverty reduction and sustainable development, infectious disease control will benefit from an approach that puts factors linked to physiology, health systems and environment together in a comprehensive, unified view of risk [45].

Because the prevalence of diabetes and *Mycobacterium tuberculosis* infection both increase with age, there is greater value in linking control efforts in ageing populations. Similarly, there is a growing number of older HIV-positive people on long-term antiretroviral treatment who have an elevated risk of metabolic, cardiac, renal, gastrointestinal, neurological and psychiatric diseases. Greater longevity also underlines the question of how to prevent cancers linked to infections that have been latent for years or decades, notably liver cancers associated with hepatitis B (preventable by vaccination) and hepatitis C viruses (treatable with drugs; see also §6) [46,47].

## 4. Infectious diseases and urban living

Since the first decade of this century, the majority of people in the world have been living in urban areas [48]. Forecasts suggest that 70% of people will inhabit towns and cities by 2050 [34]. Infectious diseases have long been regarded as a penalty for living in large, crowded populations. In fact, urbanization offers the chance—if it can be taken—to make use of better infrastructure and easier organization, so as to offset the epidemiological risks of high-density living [34].

**Figure 5.**
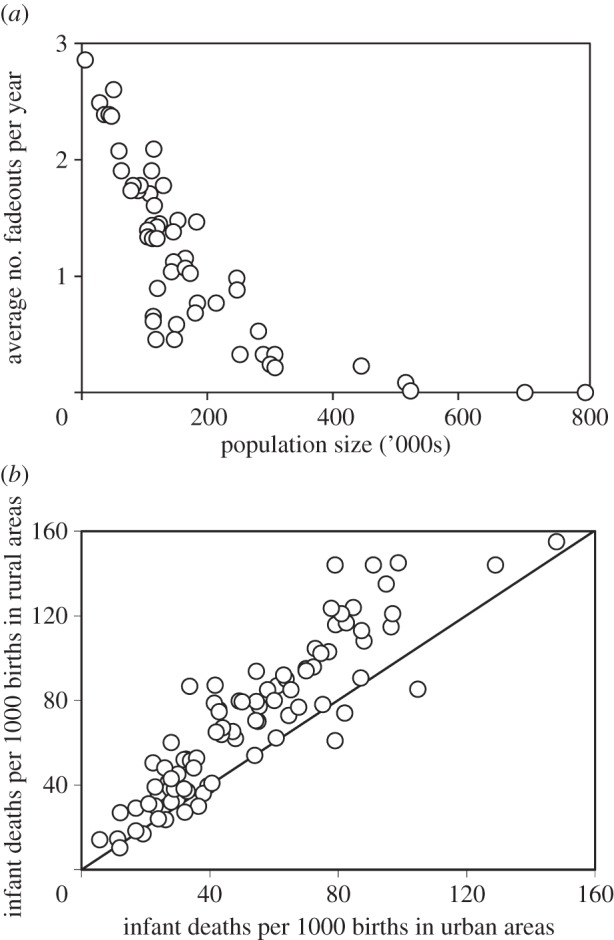
Infectious diseases in urban areas. (*a*) Annual number of fadeouts of measles (three or more consecutive weeks without a notified case) in relation to the population sizes of 54 towns and cities in England and Wales in the pre-vaccination era [49]. (*b*) Infant mortality in urban and rural areas of 90 countries worldwide. Most points lie above the diagonal line marking equal mortality in urban and rural areas, indicating that death rates tend to be higher in rural areas [50].

The urban penalty comes in two forms. The first is the price of living in urban slums, where poor housing, hygiene and sanitation expose residents to a diversity of air-, water- and soil-borne pathogens. The second is more explicitly linked to population size and density. Directly transmitted, immunizing infections can persist only in large populations—specifically in populations that generate enough susceptible individuals (mainly newborn children) to maintain transmission. Thus, measles in England during the pre-vaccination era regularly disappeared from cities with less than 500 000 people (the ‘fadeouts’ in figure 5*a*) [49].

Despite these potential risks, health is generally better in urban than rural areas worldwide. The comparative health advantage of urban living is revealed in lower infant mortality rates (figure 5*b*), which are accompanied by lower fertility rates [50]. The rural–urban difference exists because cities do usually offer better hygiene, sanitation and nutrition, and easier access to contraception and vaccination services [51–53]. In general, higher mortality is a consequence of poverty: while half the world's population lives in urban areas, less than a quarter of extremely poor people are city dwellers [54]. Health in urban and rural settings is, however, strongly tied within countries, hence the overall correlation in figure 5*b*. Urban and rural areas are linked in development.

Although these aggregate statistics favour towns and cities they mask a key feature of today's urban penalty—inequality. In terms of health services, and consequently health, the children of both rich and poor families gain from urban living, but the rich gain more. Vaccination coverage among the poorer inhabitants of cities is usually lower than among the wealthier inhabitants [53,55]. Demographic and Health Surveys (DHS) show that the ratio of child mortality (under 5 years) in the poorest 20% of families to that in the richest 20% is usually higher in urban than rural areas [50].

But while urbanization magnifies the disparities in child survival in many countries, it does not do so everywhere. Among the exceptions revealed by DHS data are Bolivia, the Dominican Republic, Egypt, Indonesia, Morocco and Peru [50]. These exceptions make plain the choices for urban inhabitants: city living can be markedly better, not just on average, but for all members of the new urban majority.

## 5. Pandemics and international health

**Figure 6.**
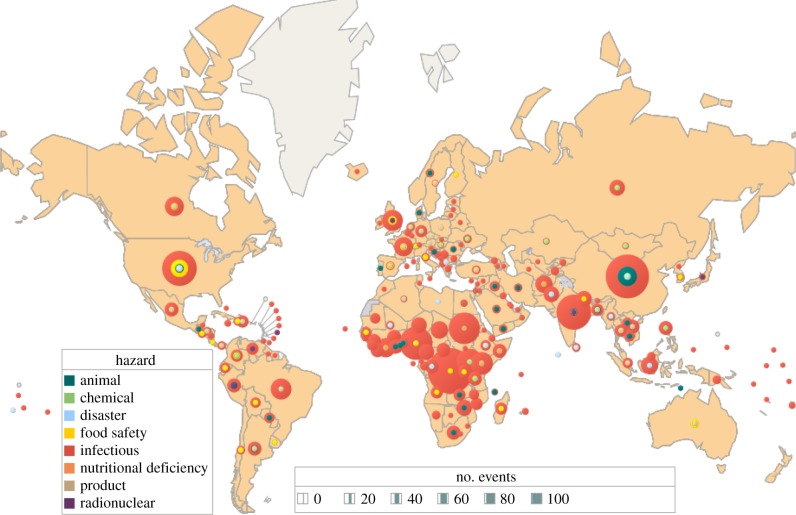
A total of 2797 international health hazards by type and country, January 2001–September 2013. Eighty-four per cent were outbreaks of infectious diseases. Unpublished WHO data (2013).

The ‘Spanish flu’ pandemic of 1918–1920 has a permanent legacy: the threat of another worldwide influenza pandemic that could kill tens of millions of people. The death toll attributed to that H1N1 virus was of the order of 50 million people. By contrast, the 2009 swine flu pandemic, also due to an H1N1 virus, killed an estimated 280 000 people, within the annual range for influenza (250 000–500 000) [56]. The 2009 influenza pandemic was just one among many unpredictable international health hazards (figure 6). Preparation in the face of uncertainty demands vigilance coupled with a rapid and internationally coordinated response [57]. This is the purpose of the International Health Regulations, a legally binding instrument for its 194 national signatories [58].

The scope of the current version, IHR (2005), and the timing of its introduction, were influenced by the emergence in China of the coronavirus that caused severe acute respiratory syndrome (SARS) in 2003 [59]. It is now mandatory to report to WHO any case of SARS, plus smallpox, polio and any new viral subtype of human influenza. According to IHR (2005), WHO must also be notified of other infections that may constitute public health emergencies of international concern, following an assessment of their public health impact. These include, but are not limited to, cholera, pneumonic plague, yellow fever and viral haemorrhagic fevers, including Ebola, Marburg and Lassa. However, the scope of the new regulations is wider and includes events other than infections, such as chemical spills and nuclear accidents (figure 6).

Do the regulations work? The global influenza pandemic of 2009 has been the severest test to date. A review of the way IHR (2005) operated during the pandemic found that the world was better prepared than ever before, but not yet equipped to deal with a severe pandemic, or indeed any large, sustained threat to public health globally [60]. Clearly, the International Health Regulations are a work in progress. It is equally clear, however, that as nations gradually build capacity for detection, rapid notification and response, international legal instruments such as IHR (2005) will have an ever more important role in health and development. By drawing on the experience of managing specific outbreaks of infectious diseases, the world's population will be better protected against international health threats of all kinds.

## 6. Expanding immunization

Smallpox is still the only human disease to have been eradicated by vaccination, the last case having been reported in Somalia in 1977. Not until 2011 was an animal pathogen also eradicated by vaccination—the morbillivirus causing rinderpest in cattle [61,62]. In the drive to eradicate certain infections, the critical question now is whether polio too can be eliminated from the last three countries in which there is persistent transmission of the virus—Afghanistan, Nigeria and Pakistan. The answer will either raise or lower expectations for eradicating other vaccine-preventable diseases, including measles [63,64].

**Figure 7.**
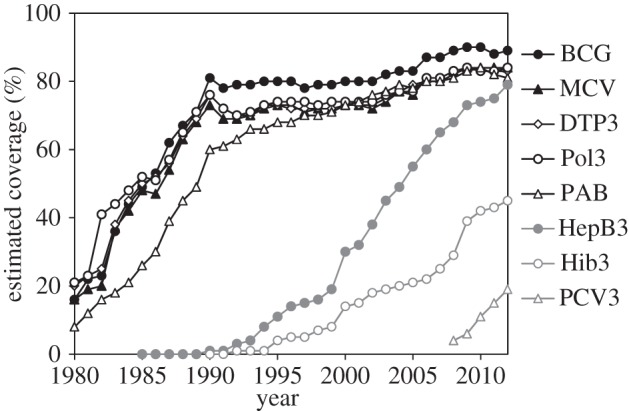
Worldwide coverage of eight vaccines used in the Expanded Programme on Immunization, 1980–2012 [66]. BCG, Bacille Calmette Guérin vaccine for TB; DTP3, third dose of diphtheria toxoid, tetanus toxoid and pertussis vaccine; HepB3, third dose of hepatitis B vaccine; Hib3, third dose of *Haemophilus influenzae* type B vaccine; MCV, measles-containing vaccine; PAB, protection at birth from tetanus; PCV3, third dose of pneumococcal conjugate vaccine; Pol3, third dose of polio vaccine.

But most vaccination programmes are not eradication campaigns [65]. Rather, their focus is on minimizing the number of cases and deaths through routine immunization. The main aim of the Expanded Programme on Immunization (EPI), established in 1974, has been to ensure that children everywhere receive life-saving vaccines [17]. During the 1970s and 1980s, vaccination coverage against acute infections of childhood, including TB, measles, diphtheria, tetanus and pertussis, increased markedly (figure 7). More recently, the coverage of several new vaccines has expanded under EPI—*Haemophilus influenzae* type b and *Streptococcus pneumoniae* (pneumococcus) vaccines to prevent pneumonia and meningitis, and rotavirus vaccine to prevent severe diarrhoeal disease.

The goal of protecting all children from acute infections is unfinished business. Nevertheless, recognizing the demographic and epidemiological changes taking place in populations worldwide, the scope of immunization programmes is also beginning to change, expanding from early childhood to vaccination through the whole life course [67].

The expansion is taking four directions. The first is to improve vaccination coverage for infants and adolescents against chronic infections, especially hepatitis B and human papilloma virus. These are causes, respectively, of liver and cervical cancers. The second is to vaccinate older children, adolescents and adults to compensate for the limited duration of protection gained from infant vaccination (e.g. pertussis), or for missed vaccination in infancy. The third is to protect the growing number of elderly people with weakening immune systems against life threatening infections, such as influenza.

The fourth is to carry out selected campaigns of mass immunization (besides that for polio). Mass vaccination against yellow fever is being reinstated after a resurgence of cases in several parts of Africa [68]; Ethiopia, for example, vaccinated everyone in six districts where yellow fever cases were discovered during 2013 [69]. In addition, given a newly developed vaccine against an infectious disease that affects children and adults, mass immunization would be the best way to gain maximum, immediate benefit for whole populations [23,24]. Thus, population-wide immunization against meningitis A in the African Sahel, with new vaccine PsA-TT, targets everyone aged 1–29 years [18–20].

For TB, mass immunization with a new vaccine would hold great appeal, especially if it could be given both to uninfected people and to those carrying latent infections. Vaccination to stop progression from latent infection to active disease would be an alternative to mass preventive drug therapy, which cannot yet be carried out at national scale. As a tool to neutralize the reservoir of latent mycobacterial infection in ageing human populations, mass vaccination would give real hope of achieving widespread TB elimination [70,71].

## 7. Towards Universal Health Coverage

Universal Health Coverage (UHC) means that everyone has access to the health services they need at a price they can afford [72,73]. In 2005, all member states of WHO made a commitment to reach that goal. The commitment was reaffirmed in 2012 through a resolution of the United Nations General Assembly (UNGA), which promoted UHC, including comprehensive primary healthcare, social protection and sustainable financing [74].

UHC is an instrument for reaching the MDGs, for alleviating poverty, and for achieving the wider results that are integral to sustainable development. The UNGA resolution sets the scene for development post-2015 by recognizing that health depends not only on having access to medical services and a means of paying for these services, but also on understanding the links between social factors, the environment, natural disasters and health.

The principal goal of control programmes for major endemic infectious diseases has been MDG 6. And the effects of programmes ‘to combat HIV/AIDS, malaria and other diseases’ have largely been measured in these terms. And yet they have contributed to achieving all eight of the MDGs, for (1) poverty reduction, (2) education, (3) gender equality, (4) child and (5) maternal health, (7) environmental sustainability and (8) building partnerships for health and development [11].

For example, the targeted control of human African trypanosomiasis and onchocerciasis has not only prevented death and chronic illness from these diseases, but also contributed to agricultural productivity. For children potentially infected with schistosomes and intestinal worms, drug treatment eliminates these infections, improves nutrition, helps to alleviate hunger and boosts educational attainment. More than 100 million infants are vaccinated with BCG each year, markedly reducing meningeal and disseminated TB as causes of child mortality. Insecticide-treated bed nets not only kill and repel mosquito vectors of malaria, but also prevent child deaths beyond those directly attributable to malaria. Intermittent preventive therapy for malaria during pregnancy is a key element both in malaria control and in improving maternal health. The same is true of HIV testing and ART delivered through antenatal clinics. The control of neglected tropical diseases contributes to reducing child mortality and improved maternal health. And better health for women promotes gender equality and female empowerment.

Infectious disease control programmes also help to build health systems [75]. For instance, the large-scale delivery of single-administration medicines by non-medical personnel (teachers, volunteers and community drug distributors) for the control of neglected tropical diseases [76] is an innovative approach in primary healthcare, based on practical and socially acceptable methods [77]. Similarly, the supervision and treatment of cohorts of TB patients on long-term therapy has inspired a similar approach for chronic, non-infectious diseases such as diabetes [78].

While infectious disease control programmes have been improving the coverage of health services, they have only recently begun to embrace the concept of UHC. Relatively little attention has been given to the provision of care within health services, notably in providing financial risk protection. Here, the challenge is to ensure that general schemes for financial risk protection satisfy the needs of patients at risk of, or suffering from, specific infectious diseases [73]. And they must satisfy the needs of everyone at risk, including the most vulnerable. A central tenet of UHC is to ‘leave no one behind’ [79].

## 8. Conclusion

After 2015, control programmes targeted against single infectious diseases will continue to be essential, not least for responding to specific and urgent problems, including outbreaks of new pathogens and the spread of antibiotic resistance. However, in the post-2015 battle to improve public health, these focused campaigns will be fought alongside others on a broader terrain.

This review has highlighted five aspects of the widening scope of infectious disease control beyond 2015, emphasizing the opportunities that go hand-in-hand with the challenges. The opportunities are to

— identify the multiple determinants of infectious diseases, including chronic diseases as risk factors, and recognize the multiple health benefits of controlling infections;
— exploit the advantages of urban infrastructure to combat poverty, inequality and associated infectious diseases, in towns and cities where the majority of people now live;
— draw general lessons from cross-border outbreaks of pathogens to reinforce the International Health Regulations, whose remit now goes beyond the containment of infectious diseases to include health hazards of all kinds;
— reinforce the EPI by scaling up the coverage of vaccines to prevent chronic diseases, and to protect adolescents and older adults in ageing populations;
— control infectious diseases while strengthening health services, moving closer to UHC.

These five themes are neither exhaustive nor mutually exclusive. They focus on epidemiology and public health, ignoring, for example, the critical question of who will pay for the post-2015 agenda. The examples in this paper have been chosen to illustrate a general point. Since the turn of the millennium, the MDGs have provided a broad framework for the control of major infectious diseases. The post-2015 agenda will be broader still. By scanning this wider horizon now, infectious disease specialists have the chance to shape a new era of health and development.
